# Molecular Dynamics Study of Naturally Existing Cavity Couplings in Proteins

**DOI:** 10.1371/journal.pone.0119978

**Published:** 2015-03-27

**Authors:** Montserrat Barbany, Tim Meyer, Adam Hospital, Ignacio Faustino, Marco D'Abramo, Jordi Morata, Modesto Orozco, Xavier de la Cruz

**Affiliations:** 1 Translational Bioinformatics in Neurosciences, Vall d'Hebron Research Institute (VHIR), Barcelona, Spain; 2 Theoretische und computergestützte Biophysik, Max-Planck-Institut für biophysikalische Chemie, Göttingen, Germany; 3 Joint IRB (Institute for Research in Biomedicine)—BSC (Barcelona Supercomputing Center) Program on Computational Biology, Barcelona, Spain; 4 Department of Chemistry, Università degli Studi di Roma "La Sapienza", Roma, Italy; 5 Centre for Research in Agricultural Genomics (CRAG), Barcelona, Spain; 6 Departament de Bioquímica i Biologia Molecular, Facultat de Biologia, Universitat de Barcelona, Barcelona, Spain; 7 Institució Catalana de Recerca i Estudis Avançats (ICREA), Barcelona, Spain; Oak Ridge National Laboratory, UNITED STATES

## Abstract

Couplings between protein sub-structures are a common property of protein dynamics. Some of these couplings are especially interesting since they relate to function and its regulation. In this article we have studied the case of cavity couplings because cavities can host functional sites, allosteric sites, and are the locus of interactions with the cell milieu. We have divided this problem into two parts. In the first part, we have explored the presence of cavity couplings in the natural dynamics of 75 proteins, using 20 ns molecular dynamics simulations. For each of these proteins, we have obtained two trajectories around their native state. After applying a stringent filtering procedure, we found significant cavity correlations in 60% of the proteins. We analyze and discuss the structure origins of these correlations, including neighbourhood, cavity distance, etc. In the second part of our study, we have used longer simulations (≥100ns) from the MoDEL project, to obtain a broader view of cavity couplings, particularly about their dependence on time. Using moving window computations we explored the fluctuations of cavity couplings along time, finding that these couplings could fluctuate substantially during the trajectory, reaching in several cases correlations above 0.25/0.5. In summary, we describe the structural origin and the variations with time of cavity couplings. We complete our work with a brief discussion of the biological implications of these results.

## Introduction

The identification and characterization of couplings between different elements of the protein structure (residues, secondary structure elements, binding sites, etc) is a subject of particular interest in molecular dynamics studies[1–7], given their role in protein dynamics, function and regulation. For example, Fenwick et al.[6] have recently shown how the study of information transfer through correlated backbone motions increases our understanding of overall structure fluctuations in beta sheets. From a functional point of view, an interesting case is that of allosteric couplings which provide[8–13] a protein-level, function regulation mechanism by which modifications (ligand binding, residue mutation, or covalent modification) at one protein site affect the binding ability of the functional site.

Here we have focused on the couplings between cavities during the native state dynamics, studying their presence, fluctuations and structural basis. We have chosen cavities because of their functional interest: they frequently coincide with the functional site of the protein[14], they can host allosteric effector sites[15–18], and their modifications by sequence mutations are at the basis of some diseases, e.g. they explain the formation of hemoglobin polymers in the case of sickle cell anemia[19].

Our work is based on the use of molecular dynamics (MD) simulations and data, and is divided into two main parts. In the first one, we have generated two independent, 20 ns trajectories for a set of of 75 proteins, containing between 6 (cow ubiquitin) and 43 (human annexin V) cavities. In this set we have studied cavity couplings in short, stable simulations around the native state. In the second part, we have used an independent set of 41 proteins for which MD simulations of length equal to, or longer than, 100 ns were available from the MoDEL project[20]. This set has allowed us to characterize the behaviour of cavity couplings in longer dynamics. For the first part, we have found a total of 297 significant couplings distributed over 45 proteins (60% of the protein dataset). We study and explain the structure origin of these couplings; subsequently, we explore their dependence on sequence changes. We find that (i) some are conserved between orthologs, and (ii) in the case of human lysozyme, two couplings in the native protein are conserved in some of its mutants. For the second part, we found that cavity correlations fluctuated along the simulation, reaching values between 0.25 and 0.5 with some frequency. Finally, we put our results in a biological context and briefly speculate on how they suggest a simple mechanism for the appearance of allostery along evolution.

## Materials and Methods

### 1. Protein datasets

#### 1.1 Set of 75 proteins with MD simulations generated for this work

This dataset (which we call D75) was obtained following a protocol designed to ensure structure quality, and the presence, for each human protein chosen, of at least one ortholog from a different species. We describe below the protocol followed.

First, we used InParanoid[21,22] to obtain a list of ortholog pairs between human and other species, restricted to those cases for which an X-ray structure was available for both species. The sequences used for this comparison were extracted from SwissProt (v. 52.5) [23,24], removing cases for which the orthology relationship was unclear. Second, we excluded NMR and modelled structures, structures with bound nucleic acids or polysaccharides, mutants and cases with model fragments, as well as structures with resolution lower than 2.5Å. When several PDB[25] files were available for the same species we kept the version with better structural quality parameters (resolution and R-factor). Additionally, at this stage we checked the sequence identity between the PDB[25] and SwissProt/UniProt[23,24] versions of the protein, to avoid identification errors. Third, for each ortholog pair, and to ensure that the PDB structures chosen for each species corresponded to the same protein region, we performed a structural alignment using MAMMOTH [26]. At the end of this pipeline, we obtained a total of 83 proteins (36 from human and 47 from 11 different species). Note that quality analysis of the MD simulations (using coordinate rmsd vs. time plots plus manual analysis) reduced this number to 75 proteins (see next section below). From a structural point of view, it must be noted that 63 of the proteins chosen were single-domain or corresponded to single-domain parts from larger polypeptides, 12 had more than one domain. The 75 proteins were distributed over the three most populated CATH[27] classes (Mainly Alpha, Mainly Beta, and Alpha Beta) and sampled 12 CATH architectures (S1 Table).

To validate our results in the ubiquitin case, we retrieved from the PDB four versions of ubiquitin's structure that also comprise its solution dynamics[28–31]. The latter is represented by an ensemble of 128, 116, 301 and 144 models, for the 1XQQ, 2K39, 2LJ5 and 2NR2 versions of ubiquitin, respectively.

#### 1.2 Set of 41 proteins with MD simulations retrieved from the MODEL project

To characterize cavity couplings in more extended dynamics, we used a set of 41 proteins (that we call D41) from the MoDEL project[20]. Of these, 36 had simulation lengths of 100 ns (S2 Table), and 5 had simulation lengths around one microsecond (1ubq, 1000 ns; 2gb1, 1000 ns; 1kte, 998 ns; 1opc, 791 ns; 1cqy, 769 ns). From a structural point of view, all the proteins but two were single-domain, or corresponded to single-domain parts from larger polypeptides (S2 Table). They were all distributed over the three most populated CATH[27] classes (Mainly Alpha, Mainly Beta, and Alpha Beta) except one, which belonged to the unfrequent Few Secondary Structures class. The CATH architectures sampled were 12; one protein had no assigned architecture.

### 2. Molecular dynamics simulations

For each protein in D75 we produced two MD trajectories (or simulation replicas) performed using AMBER 9 [32] with explicit solvent and standard equilibration, thermalization and simulation protocols at room temperature and pressure as described in MoDEL [20]. The starting point was the known experimental structure (see S1 Table for the corresponding PDB codes) removing any bound ligand. The system was then relaxed, solvated, neutralized, thermalized and pre-equilibrated using standard procedures [20]. Subsequently, we applied an equilibration step to the resulting systems, which were allowed to relax for 0.5 ns with parm99-AMBER (P99-T3P) force field [33]. These equilibrated structures were then used as starting points for 20 ns production trajectories, performed at constant pressure (1 atm) and temperature (300 K) using standard coupling schemes [20] (the same in all cases). Replicas were obtained by considering another snapshot from the equilibration after assigning a different set of random velocities. For aldolase the simulations were extended to 100 ns to perform an additional test; for ubiquitin (PDB code: 1UBQ) one simulation was extended to 100 ns, to compare our results with experimental data.

The analyses of D75 simulations were done using one snapshot collected every 200 ps; that is, 100 snapshots for 20 ns simulations, and 500 snapshots for 100 ns simulations. The cavities studied have different sizes and happen in different environments; accordingly their fluctuations will have different time scales, i.e. there is no single time interval that would allow us to retrieve all the couplings. The 200ps value was chosen on the basis of our previous work[34], where for an analogous problem (involving correlations between SURFNET cavities) we were able to find a substantial number of couplings. For D41 trajectories, we had two sampling steps: for the 100 ns trajectories, we collected one snapshot every 200ps; for ~1 microsecond simulations, we collected one snapshot every 2000ps. That is, in both cases we worked with 500 snapshots, but they represented different timescales, depending on the overall simulation length.

Crmsd values were plotted over time, for all the simulations. These plots were used as a guide for subsequent visual inspection, to eliminate those cases with an undesired behaviour, like appearance of unphysical structures, or presence of large structural deviations from the native that could degrade the cavity definitions. When applied, elimination affected both replicas of the protein, as well all its orthologs, even if these had a normal behaviour. Only two proteins were removed at this stage: calmodulin (physically unrealistic behaviour) and focal adhesion kinase (fluctuations in the hanging N-terminal affected cavity definition along the dynamics). The final dataset was constituted by 75 proteins (34 from human and 41 from 10 different species, grouped in 27 ortholog pairs and 7 triplets).

### 3. Cavity computations

We followed our previous protocol[17] (see below), in which cavities are computed with the program SURFNET[35]. Apart from SURFNET there are different ways of identifying protein cavities: Pass[36]; Fpocket[37]/MDpocket[38]; etc. We decided to use SURFNET because it is broadly used and cited in structure/function studies and its cavities cover a large range of sizes. In addition, SURFNET has been used in many cases to support or define the structure/function analysis of MD trajectories[34,39–42]. In this sense, it is worth noting that Pesce et al.[43] find an excellent agreement between SURFNET cavities and the observation of bound Xe and butyl-isocyanide molecules in their study of myoglobin and truncated hemoglobins. Note that in the case of the extended 100 ns ubiquitin simulation, we eliminated the highly flexible C-terminal end (residues 71–76) because it may lead to the appearance of spurious cavities.

In our protocol for cavity computations, for each protein we first obtained a list of its cavities and of their contouring (or lining) atoms, running SURFNET[35] with default parameters on the relaxed PDB protein structure (see S1 Table). Second, from the set of contouring atoms of each cavity we removed those atoms that can introduce artefacts in root-mean square computations (see below) because of their arbitrary definition: CG1 and CG2 from Val, CD1 and CD2 from Leu, O1 and O2 from Asp, OD1 and OD2 from Glu, NH1 and NH2 from Arg, CD1, CD2, CE1 and CE2 from Phe, and CD1, CD2, CE1 and CE2 from Tyr. The resulting atom list was characteristic of the cavity and was used in all the remaining computations (particularly crmsd) involving that cavity. Finally, from the set of cavities, we eliminated those with less than 30 atoms and/or less than 5 different residues.

Equivalent cavities between orthologs were defined as those with a maximum coincidence in their contouring atoms after structure alignment with MAMMOTH [26]. In accordance with Panjkovic and Daura[18], who used a different approach, we found that a substantial number of conserved cavities between orthologs.

### 4. Shape descriptors for cavities

Before starting to look for cavity couplings, we need to see how individual cavities fluctuate along the dynamics. We can do this in many ways, for example by looking at the variation in cavity volume, or accessible surface, or atom or residue locations/distances, etc. We decided to focus on a shape descriptor, because shape is an important component in protein-substrate recognition[44,45] and protein-protein interactions[46].

Following our recent work[17], we characterized cavity shape variations (relative to the experimental structure) along the dynamics using coordinate root-mean-square deviations (crmsd). Crmsd is a widely used shape descriptor that has been utilized to characterize functional site (FS) structure changes in MD simulations[34,47–49], in structure/function analyses[50,51], in comparative modeling studies[52], etc. In addition, it provides a direct measure of the size of the conformational space explored by the structures or sub-structures studied[53].

**Fig 1:**
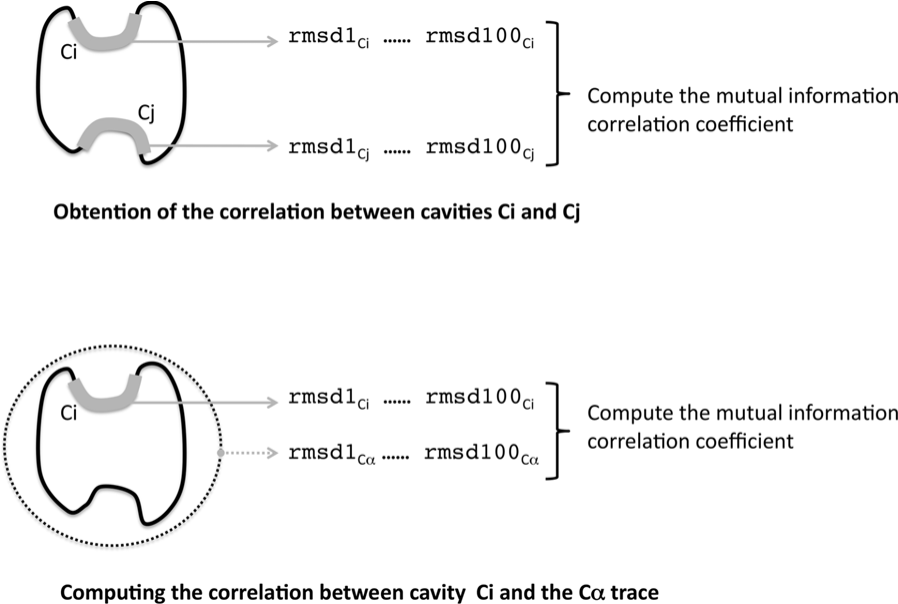
Characterization of cavity couplings. In this work cavity couplings correspond to pairs of cavities that are correlated along the dynamics. The correlations are mutual information-based and, when statistically significant, are considered to indicate the existence of a cavity coupling (see Materials and Methods). The upper half of the figure illustrates how cavity correlations were computed using the cavities' crmsd values at the different snapshots: crmsd1Ci refers to the crmsd value obtained between the set of atoms defining the i-th native cavity Ci and the first snapshot of the trajectory, crmsd2Ci for the second snapshot, etc; crmsd1Cj values have an analogous meaning for cavity Cj. The lower half of the figure shows how we computed the correlation between cavities and the Ca trace (used to understand the origins of cavity couplings, see text) along the dynamics: crmsd1Ci, crmsd2Ci, etc, have the same meaning as before, and crmsd1Ca,…, crmsd100Ca refer to the values of the Ca-trace crmsd for the 100 MD snapshots. For both the upper and lower figure halves, in the end we obtained a list of 100 crmsd pairs, which were subsequently used to compute the mutual information-based correlation between them.

Crmsd was computed after least-squares superposition of the structures compared, using the standard Kabsch algorithm[54], which eliminates the translation between structures and superposes them applying the optimal rotational transformation between them. Crmsd is then obtained using: [(1/N).∑_j_d_j_^2^]^1/2^, where N is the number of superimposed atom pairs, the subindex j varies between 1 and N, and d_j_ is the distance between the j-th snapshot atom and its equivalent in the relaxed PDB structure. To characterize the fluctuations of a given cavity along the dynamics, the crmsd computation was performed for each snapshot, superposing the cavity's conformation in that snapshot to its conformation in the relaxed PDB structure, which was always used as a reference. This gave a list of 100 crmsd values for each cavity (Fig. 1) that is at the basis of subsequent computations.

The same procedure was applied to compute the Ca-trace crmsd, although in this case all the Ca atoms of the protein were used for the alignment.

### 5. Correlations between cavities

#### 5.1 Identification of significant correlations in whole trajectories

The first half of this work was carried using D75. It was devoted to explore the presence of cavity couplings in the dynamics of proteins near the native state, using 20 ns simulations. Couplings were obtained using the protocol described in this section (see below and Fig. 1). It is important to note that they are computed for a set of snapshots; they are not a property that can be independently measured for every single snapshot like, for example, an interatomic distance. For a given protein, the *a priori* number of cavity couplings is high (e.g. if the protein has 10 cavities, there are 45 possible couplings); of these, some may be weak or spurious. For the set of D75 simulations, we decided to focus on those couplings that were more prominent relative to the background noise, and that we call **C**avity pairs with **S**ignificant **C**orrelations (CSCs). These were obtained using the following three-step protocol.

**First**, for every cavity found in a protein we computed the crmsd between its conformation in the native structure and its conformation for each of the 100 snapshots of the MD simulation, thus obtaining a set of 100 crmsd values per cavity (Fig. 1). Then, for every possible pair of cavities from the same protein, we computed the mutual information-based correlation [55,56] between the crmsd set of each cavity in the pair, and also computed its corresponding p-value, which is the probability of observing the value of the correlation by chance (from the probability distribution under independence conditions, obtained after computer-generating 10^5^ random samples). This step was repeated for the two MD simulation replicas available per protein. This correlation computation is analogous to the approach used for the identification of standard dynamical cross-correlations (Hünenberger et al., JMB, 1996), although instead of coordinate fluctuations we obtain cavity fluctuations, and also provide their p-values.

**Second**, we adjusted the p-values (FDR-adjusted p-values; see "False discovery rate (FDR)" section below) [57] to take into account the fact that the number of possible cavity-cavity correlations may be substantial. We then discarded any cavity pair having a correlation with an FDR-adjusted p-value higher than 0.05; this meant that an expected 5% of the remaining cavity pairs had correlations due to chance fluctuations.

**Third**, we applied a more stringent, and qualitatively different, filter by imposing that any surviving cavity pair must be observed in both simulation replicas to be considered a CSC. This step is really stringent and reduces the expected proportion of spurious correlations below 5%, although we do not know the size of the reduction (this would require an unfeasibly high number of MD simulations to establish). This last step was not applied to the cases for which only one trajectory was produced, e.g. in the case of ubiquitin, for which a single 100 ns simulation was used for the comparison with experimental data.

Other options are available for unveiling couplings[58,59], although they are more aimed at finding allosteric couplings, rather than the general ones considered in this work.

Identifying conserved correlations between orthologs. Given a pair of orthologs, we looked for conserved correlations between species. A correlation between a pair of cavities was defined as conserved when each cavity in the human pair matched a cavity in the other specie's pair, after aligning the structure of both orthologs with MAMMOTH [26].

#### 5.2 Fluctuations of cavity correlations in long MD trajectories

**Fig 2:**
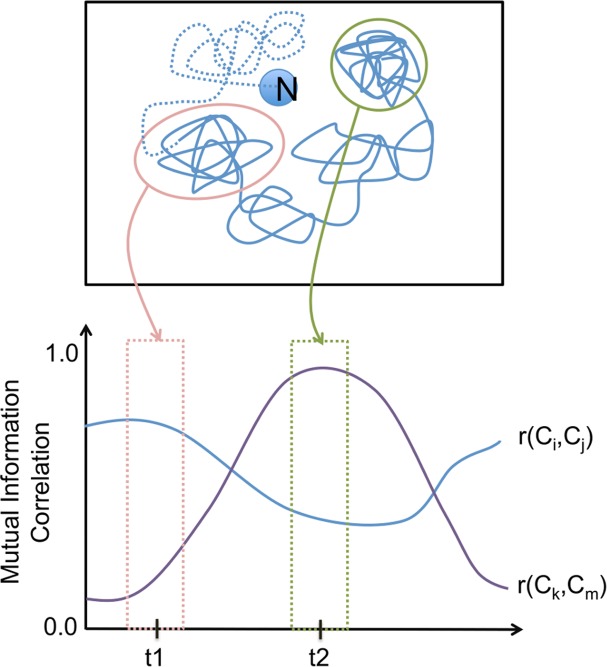
Conformational space and cavity couplings. The figure illustrates the fact that during its native dynamics, a protein may populate regions of the conformational space where a given cavity pair may have different correlations. When the protein visits the region encircled in pink (simulation time≈t1), the cavity pair (C_i_, C_j_) has higher correlation than the pair (C_k_, C_m_). The situation is reversed when the protein visits the region encircled in green (simulation time≈t2), where the pair (C_k_, C_m_) has the highest correlation.

**Fig 3:**
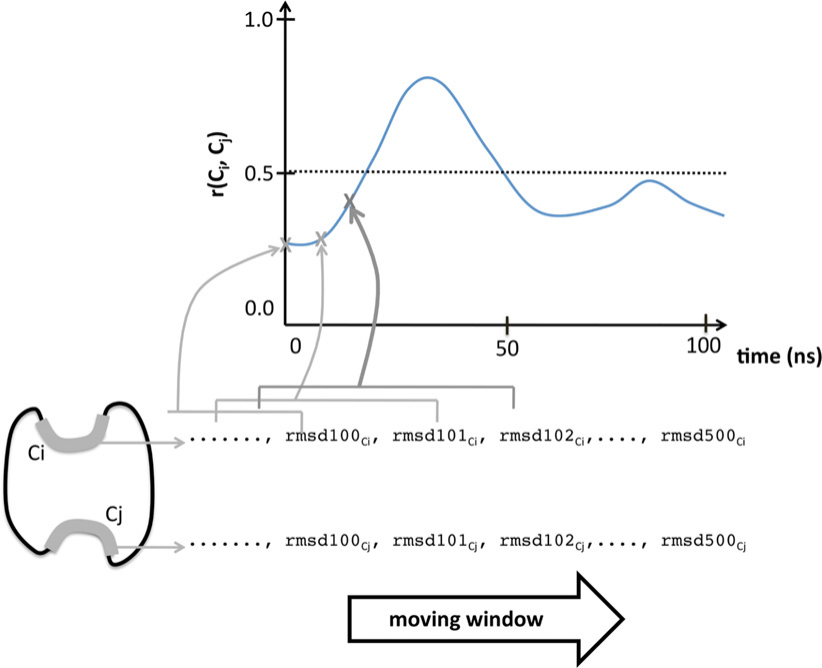
Cavity couplings along time. Correlations between cavities, computed as explained in Fig. 1A, provide a measure of their coupling. To explore how this correlation varies over time, we have used a moving window; at each position of the window we have obtained the value of the correlation and represented it, as a function of time.

In the second half of our work, carried using D41, we explored the presence of couplings along trajectories larger (simul.time≥100 ns) than those obtained for D75 (simul.time = 20 ns). In this new context, we did not apply the previous protocol as it had been designed for the analysis of simulations as a whole, not to address the time-dependence of couplings. In long simulations, the protein is more likely to populate different regions of the conformational space of the protein, where the cavity couplings may vary (Fig. 2). To represent this scenario, we decided to slide a window along the trajectory and, at each position of the window, compute the correlation for each cavity pair, using all the snapshots comprised within the window (Fig. 3). In this case we used c3net, the fast R implementation of the mutual information correlation[60].

**Fig 4:**
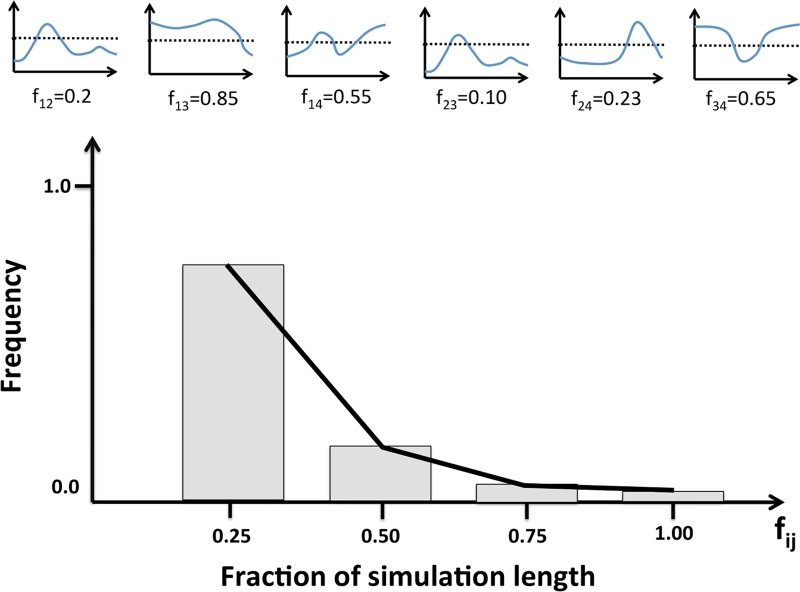
Persistence along time of the cavity correlations in a protein. The figure illustrates how we turned the correlation vs. time plots in Fig. 3 into a simpler representation allowing to summarize in a single plot the data for all the cavity pairs in a protein. The small graphs in the upper part of the figure represent the variation of cavity correlation with time for each of the cavity pairs in a protein: 1–2, 1–3, 1–4, 2–3, etc. For each of them we computed the fraction of time (f_ij_, for pair C_i_-C_j_) for which the correlation was above a given threshold. Two different values of the threshold, 0.25 and 0.5, were tried in our analyses. Finally, we represented the distribution of these f_ij_ values using a standard frequency histogram.

With this approach, we could obtain a general idea of the fluctuations of correlations in a trajectory. To this end we defined an arbitrary threshold of 0.5; then, for each cavity pair (C_i_, C_j_), we computed the fraction of simulation time, f_ij_, for which the correlation was above this value. Finally, for each protein we built a histogram with the f_ij_ values for all cavity pairs in the protein (Fig. 4). We repeated this computation using a threshold of 0.25 instead of 0.5, to explore how the trends varied.

For the computations in this section, window size was set to 20 ns, to allow a simple projection of the results in the first half of the work (obtained for 20 ns trajectories). However, similar results were obtained using other window sizes (S1 Fig.).

### 6. Explaining cavity couplings in terms of global structure changes

To see whether CSCs were related to overall structure changes along the MD trajectory, we computed the correlation between cavity and Ca crmsds (Fig. 1). Cavity crmsd (see above) reflects cavity structure/shape changes, while Ca crmsd (see above) reflects overall structure changes. Significant values of the correlation were taken as suggestive of a relationship between cavity and global structural changes.

### 7. False discovery rate (FDR)

When many statistical tests are used in a specific problem, e.g. when looking for candidate genes in microarray experiments, standard p-values are replaced by adjusted p-values, in which the effect of the number of tests is taken into account. There are different ways to produce these adjusted p-values, here we have used the approach proposed by Reiner et al. [57] which is based on the control of the false discovery rate, that is, on controlling the rate of false identifications.

### 8. Lysozyme simulations

To extend some of our analyses we ran an additional set of MD simulations for lysozyme. The MD protocol followed was the one described in the above section "2. Molecular dynamics simulations"; the analyses of the MD trajectories, like CSC computations, etc, were done according to what is described in the corresponding *Materials and Methods* sections.

For human lysozyme, we ran MD simulations for its native structure (PDB code: 1REX; chosen because of its high resolution, 1.38Å, and low observed R-merge: 6.9%)[61] and 47 mutants (see list in S3 Table) that cover a wide range of sequence modifications and physico-chemical/structural effects[62–71]. We ran 2x20 ns simulations per lysozyme.

### 9. Overlap between correlated cavities and allosteric couplings

To check whether our CSCs overlapped with allosteric couplings, and to which extent, we explored the literature and the Allosteric Database[72] (ASD, version 2.0). The information retrieved shows that experimental data were available for nine proteins: aldolase[73,74], annexin V[75–78], transferrin[79–82], retinol-binding protein[83–86] (RBP), purine nucleoside phosphorylase[87,88] (PNP), heat shock protein HSP90[89], cyclophilin A[90–92], interleukin-1alpha[93,94], and Awd nucleotide diphosphate kinase[95]. When comparing functional site (FS)/allosteric site (AS) annotations with our CSC data, we found several cases where the regions linked by both couplings overlapped: one cavity in the CSC would involve a number of FS residues and the other cavity would involve a number of AS residues (see below).

## Results/Discussion

The main goal of this study is to describe and characterize the presence of spontaneous couplings between cavities in proteins. To this end, we have divided our work in two parts: (i) analysis of a set of 2x20 ns MD simulations of 75 proteins (from 11 species, including human, see S1 Table) performed for this article; and (ii) analysis of a set of 41 MD simulations retrieved from the MoDEL project (S2 Table; duration ≥100 ns). In the first part of the work, we restricted our study to 20 ns dynamics because when looking for the structure effects underlying correlations, the results obtained are easier to interpret. In the second part, we focused on ≥100 ns simulations to give a complementary view of couplings and their robustness along protein dynamics.

### 1. Presence of cavity couplings in 20 ns dynamics around the native state

#### 1.1 Identification of significant correlations between pairs of cavities

**Fig 5:**
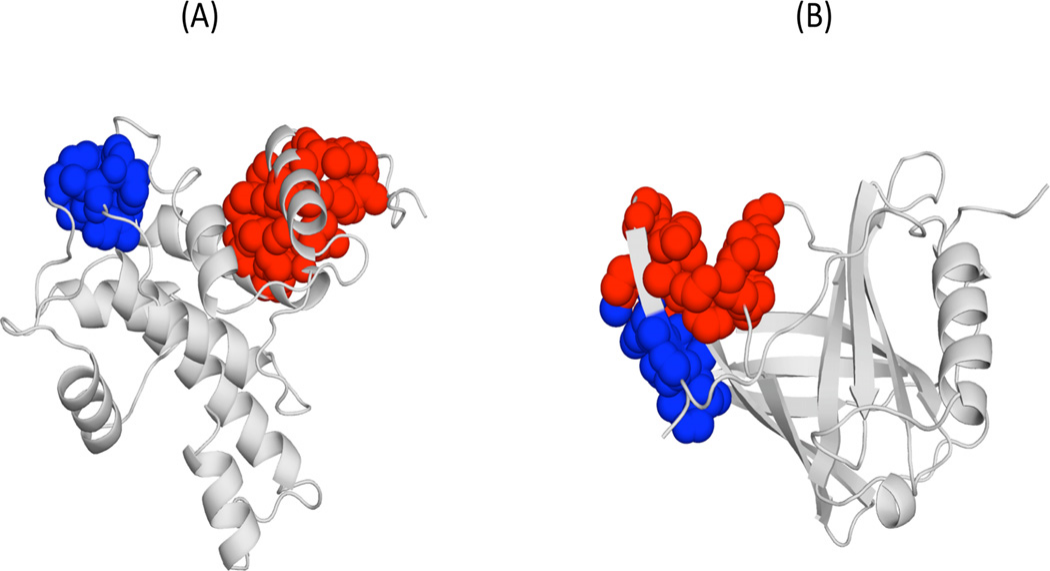
Example of cavity pairs with significant correlations (CSCs). (A) Human calpain; and (B) Human retinol-binding protein. We show the correlated cavities using a sphere representation and blue and red colours, respectively, for each cavity in the CSC; in grey we represent the protein backbone.

Using a stringent filtering procedure, which included statistical significance testing and conservation between simulation replicas, we found a total of 297 cavity pairs with significant correlations (CSCs; Fig. 5). These CSCs were distributed over 45 out of the 75 proteins studied. Human annexin and gankyrin were those with the largest number of CSCs (30 each).

**Fig 6:**
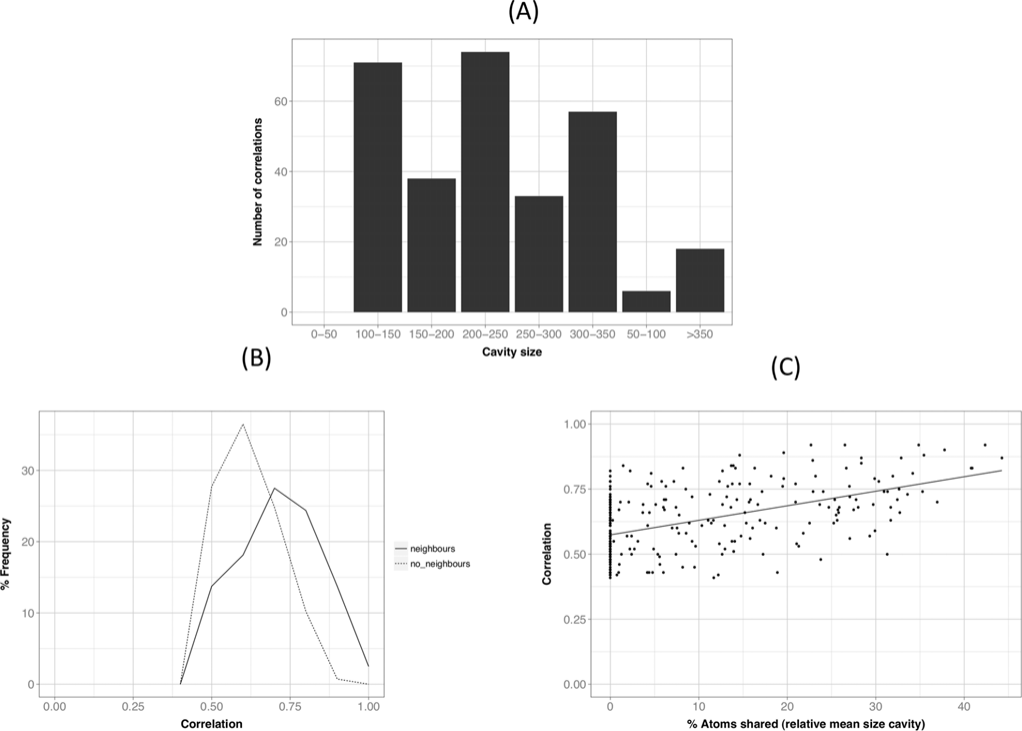
General properties of cavity pairs with significant correlations (CSCs). (A) Relationship between protein size and number of correlations. We see no clear trend indicating that larger proteins tend to host more correlations. (B) Comparison between neighbour (they share at least one lining atom) and non-neighbour (no lining atom is shared) cavities: range and frequency distribution. (C) Dependency of the size of correlation on the percentage of atoms shared (normalized to the mean size cavity for each CSC). We see a slight monotonically increasing trend, indicating that the more atoms shared, the higher the correlation.

We checked if the number of CSCs varies with protein size, but no clear relationship appeared (Fig. 6A). We then explored how these CSCs were distributed relative to two possible sources of cavity correlation (Table 1): neighbourhood (effect of atom sharing) and global structure dynamics (identified by computing the correlation of cavity crmsd with Ca trace crmsd).

**Table 1:** Distribution of the cavity pairs with significant correlations (CSCs) relative to their properties: correlation with the Ca trace and the degree of neighbourhood between the correlated cavities.

CSCs	Neighbours	No neighbours	Total
**No Cavity Corr. with Ca**	74 (11)	73 (3)	147 (14)
**One Cavity Corr. with Ca**	48 (11)	39 (0)	87 (11)
**Two Cavities Corr. with Ca**	38 (2)	25 (1)	63 (3)
**Total**	160 (24)	137 (4)	297 (28)

In parentheses we show the figures for the CSCs conserved between orthologs: 14 pairs, that correspond to a total of 28 CSC cases.

Effect of atom sharing. Cavities sharing some of their contouring atoms (Fig. 5B), what we will call from now on neighbour cavities, may naturally appear as correlated along the dynamics. Quantitatively we followed a strict definition of neighbourhood: two cavities with at least one lining atom in common were considered neighbours. Of our 297 CSCs, 160 involved neighbour cavities and 137 non-neighbour ones. We checked whether neighbourhood contributed substantially to the average correlation coefficient between cavities. To this end we divided our CSC dataset in two: pairs constituted by neighbour and non-neighbour cavities, respectively. In general, the former had larger correlation coefficients (Fig. 6B, p-value of the Kolmogorov test = 1.8x10^-8^), although there was a clear overlap in the correlation range. This was confirmed when plotting correlation value vs. percentage of shared atoms (Fig. 6C), which showed that the expected monotonically growing trend was relatively mild, and that correlations for CSCs that involve cavities with no common atoms are important (points sitting on the y-axis). In other words, coupling of cavities happens in the absence of common atoms and can affect structurally distant cavities.

**Fig 7:**
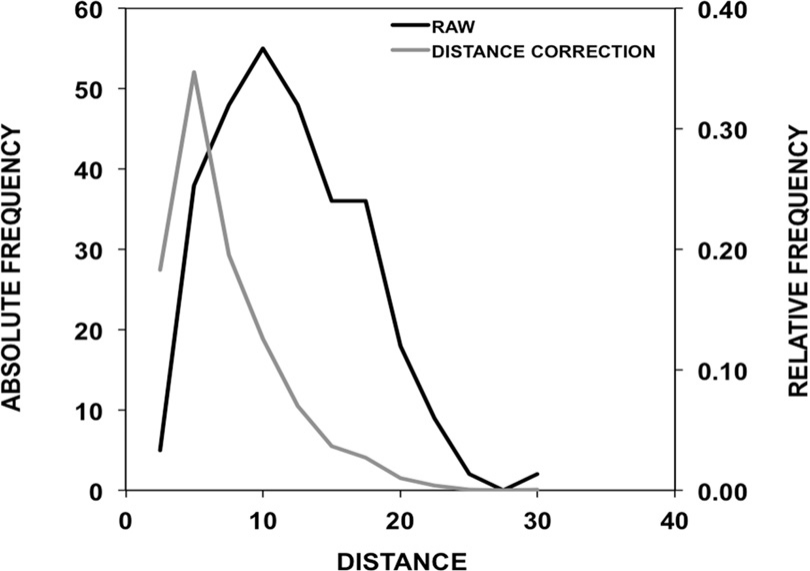
Dependence of the number of CSCs on the distance between cavities. We show two curves: in black the one corresponding to the raw number of counts (scale in the left Y-axis); in grey, we show this value normalized to eliminate the volume effect (dividing each count by the square of the distance; scale in the right Y-axis). We can see that CSCs tend to happen more frequently for close than for distant cavities.

Apart from the neighbour analysis, but related to it, we have explored how the number of CSCs depends on the distance between cavities (Fig. 7). To this end, and for each CSCs we computed the average location of the lining atoms of each cavity, and obtained the distance between these virtual points. While this analysis is limited by the fact that cavity shape is irregular, we can see that CSCs tend to be more frequent at shorter distances, an effect that becomes more clear when we normalize the data for the distance-dependent volume effect.

**Fig 8:**
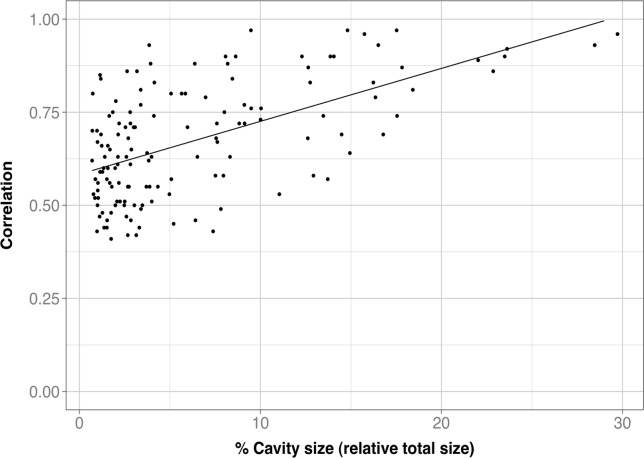
The relationship between the cavity-Ca trace correlation and fraction of cavity atoms relative to the total number of protein atoms. We can see a weak trend indicating that the bigger the cavity, the larger the correlation with Ca-trace crmsd. This is understandable, as larger cavities include among their lining atoms more Ca, or Ca-related, atoms than smaller ones.

Cavity correlation and global structure effect. To check if CSCs are related to the global dynamics of the protein [17] we explored how global structure changes correlated with cavity structure changes, as measured by crmsd (Fig. 1, and *Materials and Methods*, section 6). For 150 CSCs (Table 1) at least one of the cavities in the pair showed structure fluctuations correlating with Ca-trace fluctuations; the value of the correlation was larger for bigger than for smaller cavities (Fig. 8; R^2^ = 0.33, p-value = 2.2x10^-6^). This trend is to be expected, as the larger the cavity the larger its contribution to global protein fluctuations. Finally, we identified (Table 1) 63 CSCs for which both cavities had crmsd correlating with Ca fluctuations. This result indicates that global structure fluctuations can contribute to originate the observed CSCs.

Apart from the previous contributions to cavity couplings, pathways of residue-residue contacts could also play a role, particularly for non-neighbour cavities, as in the case of allosteric couplings [9]; this would be in accordance with the fact that CSCs tend to be more frequent at shorter than at longer distances (Fig. 7). Also in the case of surface cavities, their couplings could be modulated by the network of water molecules constituting the hydration shell, in a similar way as has been described for myoglobin[96], where water networks modulate functional properties.

In summary, 60% of the proteins displayed CSCs in their dynamics, showing that for the proteins in our dataset it is not rare to find couplings between cavities when they fluctuate around their native structure. The most frequent cause of proteins with missing CSCs is the stringency of our filtering procedure, particularly of the last step. Before applying it, the number of statistically significant cavity couplings was of 1343 for one replica and 1430 for the other. These values corresponded to 84% and 85.3%, respectively, of proteins with at least one significant coupling. After applying the last step of our protocol, we observed a drop from ~85% to 60% in the number of selected cavity couplings; the stringency of this step suggests that 60% is a lower threshold. Data from our recent work[34] in glutathione S-transferase and ectodysplasin-A support this result by showing that cavity couplings can be found in different alternative splicing isoforms of the same protein. Interestingly, Bowman and Geissler[97] have recently found that this is also the case for cryptic allosteric sites. In their study these authors have used massive MD simulations to assess the frequency of these sites in three different proteins (beta-lactamase, interleukin-2, and RNase H), finding that they are ubiquitous during the equilibrium dynamics of proteins. Considering together our data and those from Bowman and Geissler[97] a picture arises in which couplings between protein sites could be a frequent phenomenon during the equilibrium dynamics of proteins.

Additional evidence for the presence of these couplings can be obtained from four experimental studies aimed at describing the structural ensemble of ubiquitin's native state [28–31] (see *Materials and Methods*). We found that CSCs were present in the four experimental versions of ubiquitin; they were also present in a 100 ns extended ubiquitin simulation, performed with comparison purposes. Note that in this case we used the more stringent Pearson correlation coefficient, to identify cavity couplings. In addition, the number of CSCs for the simulation, 21, was within the range of values observed for the experimental models (1XQQ: 12; 2K39: 26; 2LJ5: 22; 2NR2: 25). We then compared the CSCs observed in the experimental dynamics with those observed in the simulation, to see if they were equivalent. To establish a baseline, we first cross-compared the experimental data, finding that CSC conservation varied between 3 (for the pair 1XQQ-2LJ5) and 9 (for the pairs 2K39-2LJ5 and 2LJ5-2NR2). Note that no absolute coincidence was expected, given the large size of the conformational space, even in the native state. The results of comparing the CSCs from ubiquitin's simulation and those of experimental origin gave conservation numbers (3, 5, 4 and 6) within the baseline range (between 3 and 9). In summary, the coincidence in presence, number and type of CSCs between experiment and simulation supports the common existence of CSCs.

#### 1.2 Robustness of CSCs to sequence changes

In the previous section we have seen that CSCs are present in 60% of the proteins in our dataset, and we know that these proteins may have different folds (Fig. 5; S1 Table). A question that naturally arises is whether there are conserved CSCs between similar structures or how robust are CSCs to sequence changes preserving protein structure. This question is interesting from a fundamental point of view as it relates sequence changes to protein structure/dynamic properties, and also because some couplings could have a functional role. In this section we describe how CSCs vary between proteins that differ in one or a few amino acids, and how they vary between orthologs, where sequence changes may be larger.

**Fig 9:**
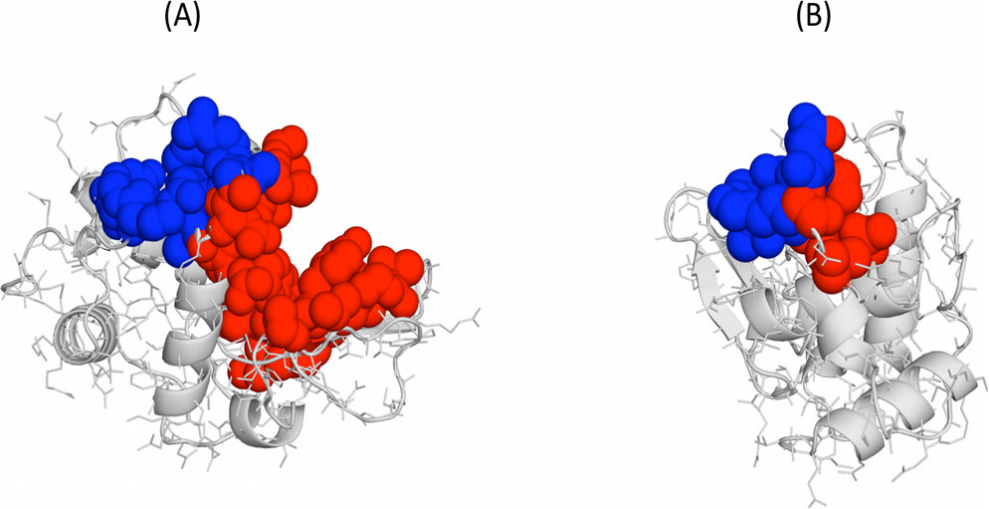
View of the two CSCs found in the native human lysozyme (PDB code: 1REX). The coupled cavities are shown in blue and red, to distinguish them. (A) The blue cavity includes the protein's functional site, pointing in this case to a possible functional relevance; (B) two minor cavities that are also coupled.

In the first case, we used a set of 47 human lysozyme mutants with known X-ray structure (see *Materials and Methods* and S3 Table). We applied our protocol for identifying CSCs (*Materials and Methods* section "5.1 Identification of significant correlations in whole trajectories") to both the native and the mutants: we ran 2x20 ns MD simulations per protein, and checked cavity coupling conservation in the mutants relative to the human protein. We found that the native lysozyme had two CSCs (Fig. 9): one involving lysozyme's functional cavity and an adjacent cavity and the second involving two smaller cavities. The first coupling was conserved in 11 mutants (23%), and the second in 4 mutants (8%), indicating that in lysozyme CSCs may survive single sequence mutations.

**Fig 10:**
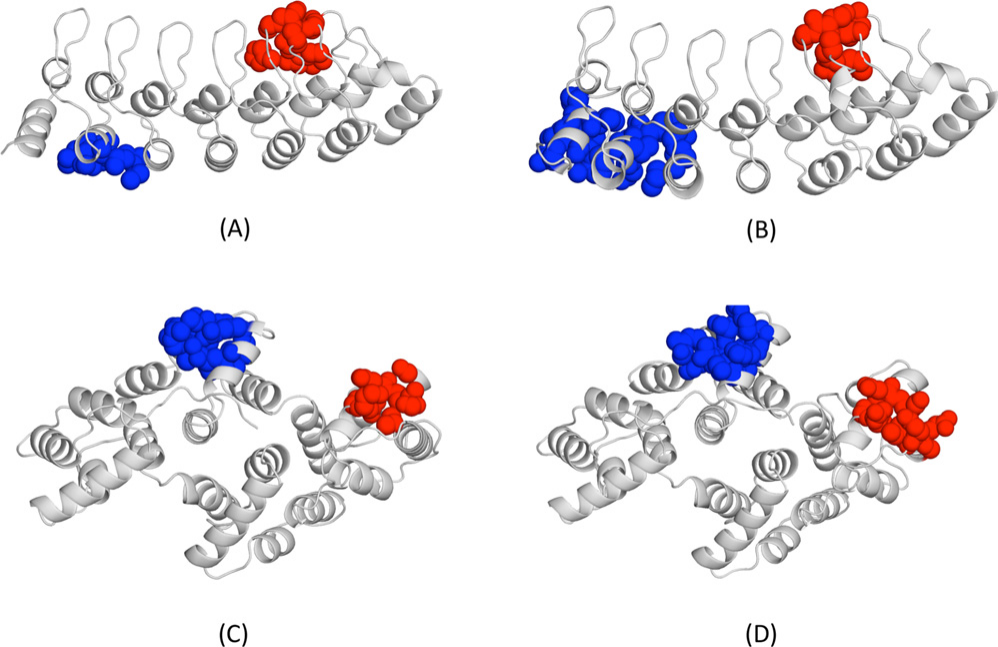
Conservation of cavity couplings between orthologs. In the figure we show two cases, where coupled cavities share no atom, that is, are non-neighbours. (A) and (B) correspond to human and yeast gankyrin orthologs, respectively; (C) and (D) correspond to in human and chicken annexin orthologs.

**Fig 11:**
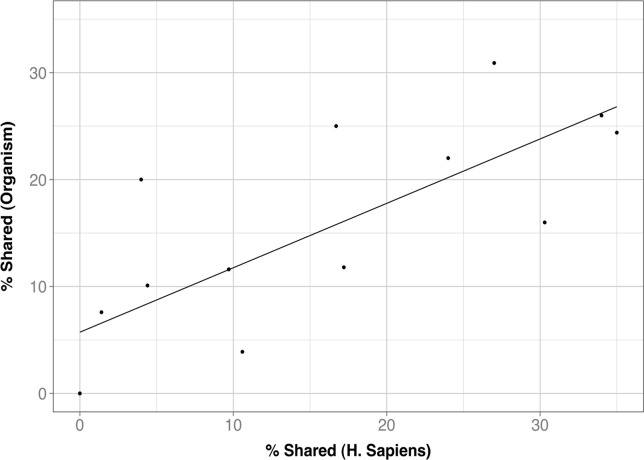
Neighbourhood conservation in conserved couplings. We can see that there is a relationship between the number of neighbouring atoms in human and in the other species, indicating that these two values tend to be conserved between species.

**Fig 12:**
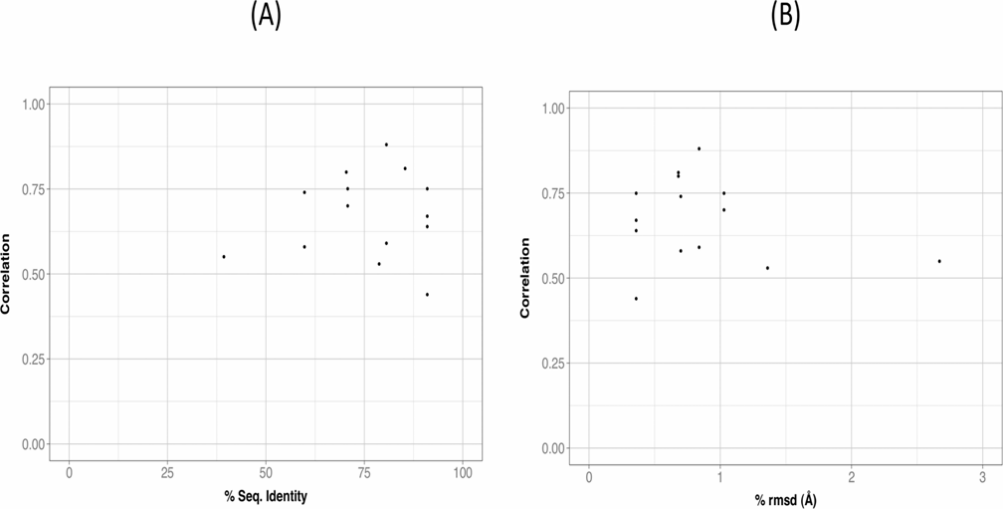
Relationship between correlation size and protein divergence. (A) sequence level, and (B) structure level. For each of the 14 pairs of couplings conserved between species, we computed the average between the human and the other species correlation (an average of two values). Then, we plotted the resulting 14 values as a function of protein sequence identity (A) and of crmsd (B). In none of these two cases we saw a relationship, suggesting that other factors may determine correlation conservation.

In the second case, we checked if CSCs were conserved between the orthologs in our dataset. In this case the sequence changes were generally more drastic than for the lysozyme mutants (sequence identity range: from 39%, Gankyrin human-yeast pair, to 98%, ubiquitin-conjugating enzyme UBC9 human-mouse pair). We found (Table 2, Fig. 10) 14 cases for which the human CSC had an equivalent in a non-human ortholog, i.e. a total of 28 CSCs; the remaining CSCs, ~91% of all those identified, were protein-specific. The lower number of conserved CSCs may result from a combination of several factors: some will be physically/biologically relevant, and some may be spurious (technical origin). Among the former, we may cite the appearance of differences, after ortholog divergence, in the delicate residue-residue contact networks coupling remote structural elements. This possibility, advanced by Livesay et al.[58] in the case of allosterism, could explain why there are so few conserved couplings formed by non-neighbour cavities in D75: the human/yeast gankyrin pair (Fig. 10A-B) and the human/chicken annexin pair (Fig. 10C-D). In front of these two cases, the higher number of CSCs involving neighbour cavities, 12, is probably due to the better conservation of neighbourhood between orthologs (Fig. 11; Pearson's correlation = 0.59, p-value = 1x10^-3^). However, while conservation of structure features is usually related to sequence identity, we found no relationship between the size of the correlation coefficient associated to CSCs and three global measures of protein divergence: percentage of sequence identity (Fig. 12A; Pearson's correlation = 0.009, p-value = 0.75), crmsd between protein structures (Fig. 12B; Pearson's correlation = 0.06, p-value = 0.42) and Kimura's distance[98] (S2 Fig.; Pearson's correlation = -0.16, p-value = 0.58), a measure of sequence divergence used in evolutionary studies. In fact, the ortholog pair with the lowest sequence identity (39.3%) had a relatively good correlation for the CSC in both ortholog pairs, 0.55 and 0.54, for human and yeast, respectively.

**Table 2:** Lists of cavity pairs with significant correlations (CSCs) conserved between orthologs.

HUMAN			SPECIES			
PDB	correlation	%shared	PDB	correlation	%shared	organism
**1hup**			**1rdo**			r. rattus
2–26	0.52	1.4	3–15	0.63	7.6	
16–28	0.77	16.7	18–21	0.71	25	
**1iu1**			**1gyu**			m. musculus
5–9	0.87	27	4–7	0.74	30.9	
**1kpb**			**6rhn**			o.cuniculus
3–4	0.46	4.4	5–6	0.41	10.1	
3–21	0.57	9.7	5–21	0.71	11.6	
6–11	0.70	34	9–11	0.79	26	
7–11	0.66	24	10–11	0.68	22	
**2ald**			**1zah**			o.cuniculus
6–12	0.59	15.9	3–13	0.66	9.9	
**2cpl**			**1dyw**			c.elegans
16–31	0.88	35	17–24	0.71	24.4	
**1j7d**			**1jbb**			s.cerevisiae
6–11	0.87	30.3	7–29	0.63	16	
5–6	0.77	4	3–7	0.62	20	
**1uoh**			**1ixv**			s.cerevisiae
24–26	0.55	0	23–5	0.54	0	
**1tbt**			**1v9e**			b. taurus
1–4	0.68	10.6	1–2	0.49	3.9	
14–36	0.92	17.2	23–18	0.84	11.8	
**1anx**			**1yii**			g. gallus
32–33	0.52	0	13–11	0.53	0	

Only CSCs for which both cavities in the pair have an equivalent in both species are listed. We also provide the percentage of shared atoms between cavities, within each species (for a given CSC, 0% means that no atom is shared between the two cavities defining a CSCs; 100% means that all of them are shared, this would correspond to equal cavities, a non-existent case). The first three columns correspond to human CSCs data, the next three correspond to the ortholog's CSCs, and the last column gives the ortholog's species.

In summary, for both the native-mutant comparisons in lysozyme and those between orthologs, we find that not all CSCs are conserved between structures with the same fold. However, we could not see a clear trend relating sequence changes and CSC survival/loss. This suggested that an additional factor could explain the pattern of presence/absence of couplings. In the next section we use longer simulations to address this problem.

### 2. Variation of cavity correlations along protein dynamics

#### 2.1 The case of aldolase

Previously, we have seen that for the 2x20 ns simulations, 60% of the proteins in D75 display CSCs. Interestingly, we have also seen that there was a large number of simulation-specific cavity couplings discarded because they did not appear as statistically significant in both simulations, thus failing to meet the last condition of our filtering procedure. Some of these couplings could be spurious and therefore correctly rejected. Others, however, may have failed to pass this filtering step because the way in which a protein travels through the conformational space may induce fluctuations in the cavity correlations (Fig. 2). To explore this possibility, we looked for the presence of fluctuations in cavity correlations over time, extending one of our simulations from 20 ns to 100 ns. Then, we used a 20 ns moving window along the trajectory (Fig. 3), computed the cavity correlations at each window location, and represented them as a function of time.

**Fig 13:**
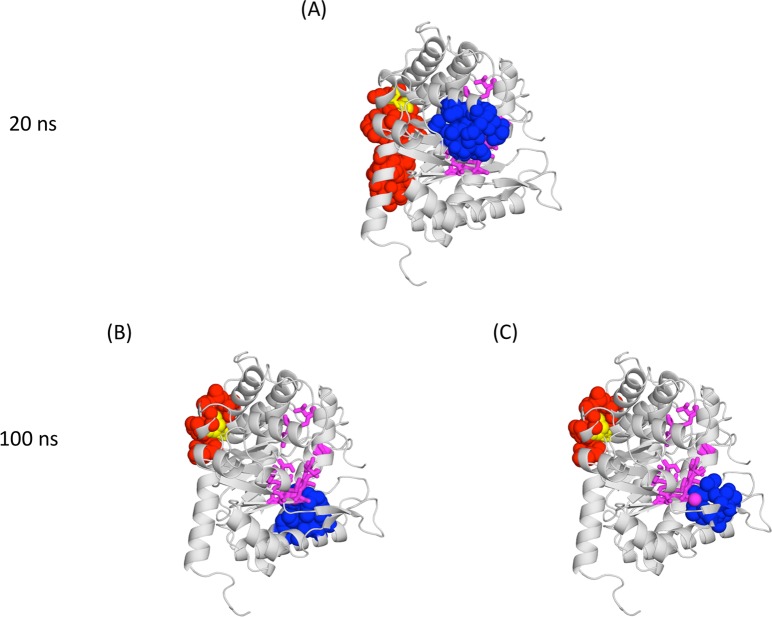
Human aldolase simulations. (A) 20 ns results, showing in magenta sticks and yellow spheres the protein binding and allosteric sites, respectively. In blue and red we show the coupled cavities including the binding and allosteric sites, respectively. In (B) and (C) we follow the same colour code, except that the data were obtained from a 100 ns simulation (obtained after extending one of the 20 ns replicas). (B) and (C) illustrate the two couplings found in this simulation.

We concentrated on a few CSCs, related to experimentally substantiated inter-site couplings. To this end we first identified the allosteric proteins in our dataset (Table 3) and kept only those having CSCs that overlapped with the functional (FS) and the allosteric (AS) sites. We imposed an additional condition: that the allosteric coupling had to be present in both the human protein and its ortholog. At this stage we were left with two proteins, aldolase and HSP90, and arbitrarily chose aldolase. For human aldolase there is a known functional coupling between two aldolase regions[73] and several CSCs (Fig. 13A) overlapped with them.

**Table 3:** Overlap between cavity pairs with significant correlations (CSCs) and known allosteric couplings.

Protein	FS residues	AS residues	human CSCs	species CSCs
**ALDOLASE**	D33,S35,S38,K107,K146,R148 E187,E189K229,S271,R303,L356	C72,C338	YES	YES
**ANNEXIN**	M28,G30,G32,K70,E72,L73 E78,G183,K186,G188,E228	W185	YES	NO
**TRANSFERRIN**	R140,K203,K293	E80,Y82,H246,K293	NO	——
**RBP**	L35	L63-D68,G92-K99	YES	NO
**PNP**	F199,E200,V216,M218,N242 V244,V259	——	——	——
**HSP 90**	D83,N51,G137,F138	K194,L220,Q287,P295	YES	YES
**CYCLOPHILIN**	H54,R55,F60,M61,Q63,N102 Q111,F113,W121,L122,H126	C51,H53,H69,K150,T151,K154	——	——
**INTERLEUKIN-1beta**	N87-K101	W118	NO	NO
**NDK**	——	——	——	NO

The residue numbers of the functional (FS) and allosteric (AS) site residues were obtained from the literature (see main body of the article). In the fourth and fifth columns we mention whether there was any CSC with a cavity overlapping the FS and the other the AS: YES (at least one CSC existed), NO (no overlapping CSC was found), dashed line (——, functional information was lacking and the comparison could not be performed).

**Fig 14:**
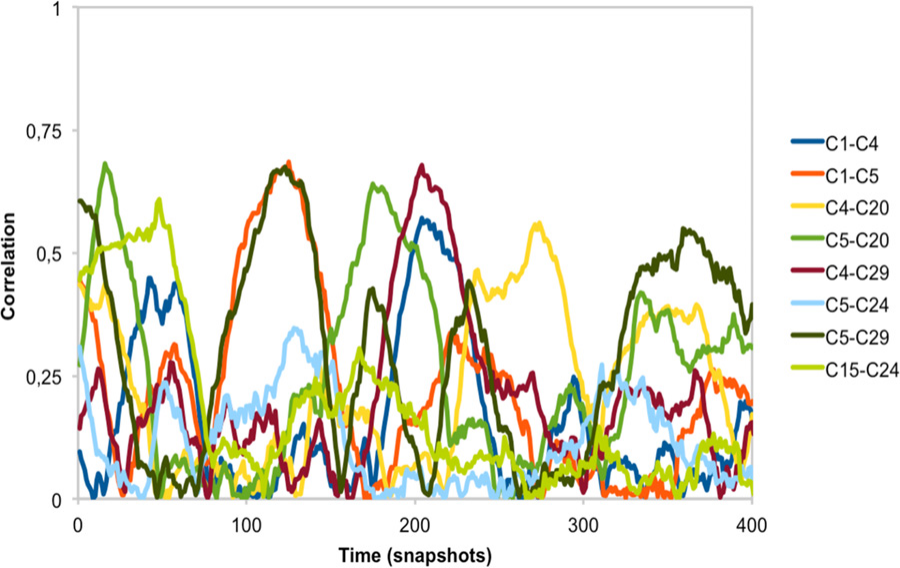
Fluctuations over time of cavity pairs in aldolase. For a set of cavity pairs overlapping the functional and allosteric sites, we plot the fluctuations over time, using the moving window approach described in the *Materials and Methods* and illustrated in Fig. 3. Each colour line represents a cavity pair, as explained in the legend. Time is represented by the snapshot number.

In accordance with our expectations, when we extended one of the 20 ns aldolase simulations to 100 ns, we identified two statistically significant couplings (involving AS and FS residues; Fig. 13B and 13C) that did not coincide with the one observed for the 20 ns simulations (Fig. 13A). When using the moving window on the 100 ns trajectory we found noticeable fluctuations in the cavity correlations (Fig. 14): a correlation could go from 0 to nearly 0.75 and back to 0. This result clarifies the picture of cavity correlations obtained from the 2x20 ns simulations, indicating the importance of taking into account the variation of couplings along the simulation. We address this issue in the next section.

#### 2.2 Fluctuations of cavity correlations along MD simulations

To see how the results for aldolase extended to other proteins we required simulations longer than the 20 ns we had generated. For this reason, we used a set of 41 simulations ≥100 ns available from the MoDEL project[20] (we call this set D41, see S2 Table). In this case, and bearing in mind the results for aldolase, we approached cavity correlations from a different angle. Rather than considering statistical significance at a single point in time (as in the analysis of the 2x20 ns simulations), we focused on their fluctuations along the dynamics. For this reason, we included all possible cavity pairs in our analysis, since their correlation could vary substantially. Technically, we followed a simple procedure: for each cavity pair we computed the fraction of the simulation length for which the correlation between cavities was above 0.5. High values of this fraction mean that during a large part of the dynamics, the correlation between the cavities in this pair was above 0.5; low values mean the contrary. This approach allowed us to simultaneously plot the data for all the cavity pairs of a protein (representing them with a single line, see Fig. 4), and for all D41 proteins at the same time. The correlation threshold value, 0.5, was chosen because it is halfway between the two values defining the mutual information correlation range (0: absence of correlation; 1: complete correlation). Also, because it is near the lowest correlation value giving significant p-values in the 2x20 ns simulations (Fig. 6B-6C). However, we reproduced all the computations with 0.25 as a correlation threshold, to explore the effect of a more permissive value, as the aldolase case (Fig. 14) shows that many cavity pairs can have low correlation values along the dynamics. (Note: we had also checked the effect of the moving window size, sampling 10, 20, 30, 40, 50, with no effect; see S1 Fig.).

**Fig 15:**
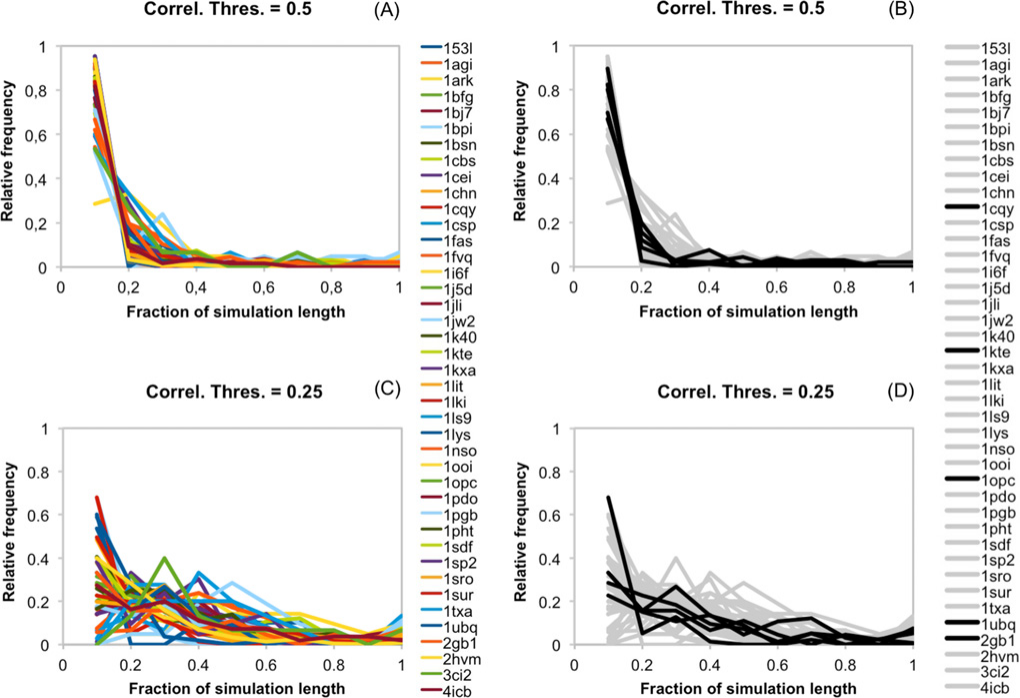
Summary representation of the persistence of cavity correlations along protein native dynamics. Each coloured line in (A) and (C) represents the distribution of the fraction of simulation time spent by the cavity pairs of a protein above a given threshold (0.5 for (A) and 0.25 for (C)). The obtention of this distribution is explained in *Materials and Methods* and illustrated in Fig. 4 for a single protein. The distinguishing feature in (B) and (C) is that we plot in grey all proteins with 100 ns trajectories, and in black the cases with simulation lengths above 100 ns.

We saw the same behaviour for essentially all proteins in D41 (Fig. 15A): a rapid decay in the fraction of cavity pairs as a function of the time spent in higher-than-0.5-correlation regions. More qualitatively: during a native dynamics the correlation of most of the cavity pairs will always, or almost always, be under 0.5. Only a few will deviate from this trend, their number becoming smaller with the time their correlation is above 0.5. This general behaviour is comparable for all the proteins, although the decay speed varies between them (Fig. 15A). When we repeated this analysis with the more permissive correlation threshold of 0.25, we found a similar result. Here, however, the average decay was slower (Fig. 15C). Indeed, it is not unusual for a protein to have cavity pairs with correlations above 0.25 during a third of the trajectory, something expected from the aldolase result (Fig. 14).

The fast decay observed in Fig. 15A explains why imposing coupling conservation between replicas is such a tough filter in the identification of CSCs: it is unlikely that a coupling has high correlation values at the same time point in two independent simulations.

Another point of interest is whether our results can change depending on the length of the simulation or, whether the results in Fig. 15A and 15C have converged. To test whether this was the case, we represented in grey the 36 proteins with 100 ns simulations, and in black the 5 proteins with simulation lengths around the microsecond. The resulting plots (Fig. 15B and 15D, for 0.5 and 0.25 correlation thresholds, respectively) indicate that 100 ns and near-microsecond simulations gave a comparable picture of the behaviour of cavity couplings along simulation time.

**Fig 16:**
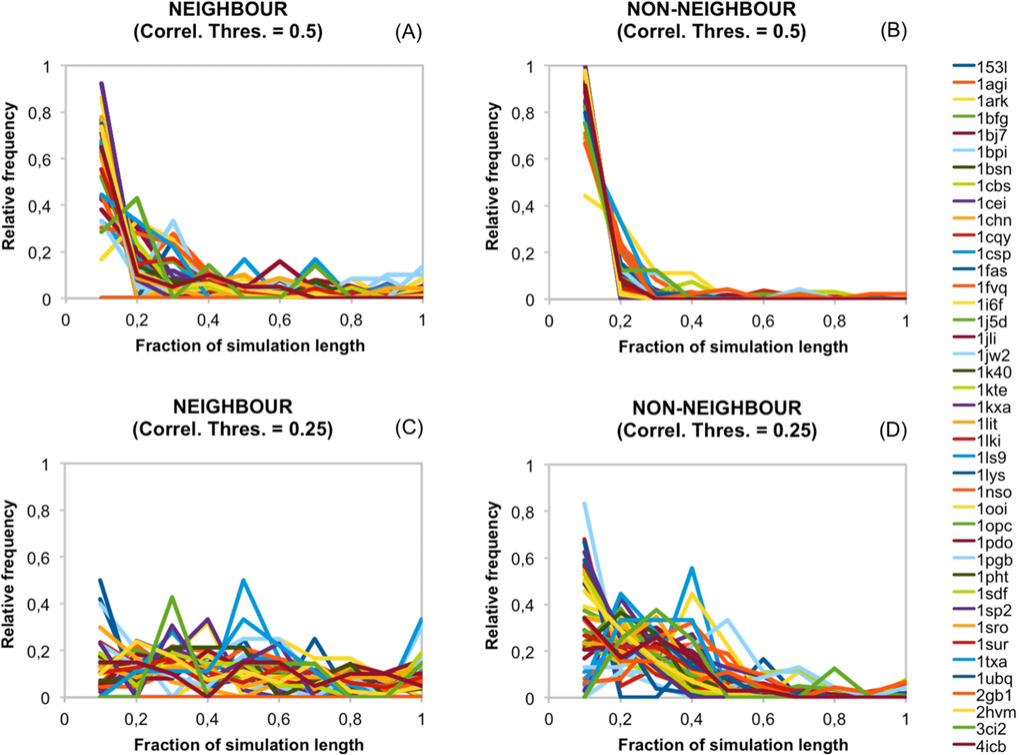
Effect of cavity neighbourhood on the fluctuations of cavity pair correlations. We divided into two the contribution of each protein to Fig. 15. (A) Represents only the correlations between neighbouring cavities (sharing at least one of their lining atoms). (B) Represents the data for non-neighbouring cavities. Again each colour line represents a protein. The two upper figures result from using a correlation threshold of 0.5; a value of 0.25 was used for (C) and (D).

Finally, we explored to which extent neighbourhood affected the fluctuations of cavity correlations over time. To this end we reproduced Fig. 15 but now separately plotting the results for neighbour and non-neighbour cavity pairs. The results, represented in Fig. 16 for both correlation thresholds, showed that pairs of neighbour cavities spent more time at correlations above 0.5 than non-neighbour pairs. The same was true when using 0.25 as the correlation threshold. This result is in accordance with the analyses of the 2x20 ns simulations pointing at neighbourhood as a component of cavity correlations.

In summary, the data in this section extend and improve the view obtained from the 2x20 ns simulations. In particular, they tell us that, for our protein dataset, couplings vary along the dynamics (Figs. 14, 15A and 15C). This behaviour depends on whether the cavity pairs involve neighbour or non-neighbour cavities, with the former surpassing more frequently correlation thresholds (e.g. 0.25 or 0.5) than the latter (Fig. 16). Finally, mention that this view of cavity coupling holds regardless of whether we consider 100 ns or >100 ns dynamics (Fig. 15), indicating a reasonable degree of convergence in our results. Note that both the data for this and the previous section were mostly obtained for single-domain proteins (S1 and S2) Tables; they must be considered with care in the case of multidomain proteins; in these proteins, cavity pairs at the boundary between domains may display unexpected behaviours.

### 3. Biological implications of the observed cavity couplings

The scenario arising from our previous sections indicates that many proteins may present cavity couplings. We do not know if these couplings are allosteric couplings. Different data indicate that some of them may be comparable. First, it has been recently shown by Long and Brüschweiler [99] that correlation coefficients, in their case between torsional angles, provide a good measure of allosteric transmission between sites. Second, in our 2x20 ns study we observed 19 proteins with at least one CSC involving the main cavity (usual locus of the functional site [14]), suggesting a regulatory role for these CSCs. Third, we found CSCs overlapping with allosteric couplings (Table 3) in some of the proteins of our dataset. And fourth, in the 100 ns simulation of aldolase we saw that cavity pairs overlapping with the functional and allosteric sites had non-zero correlations (Fig. 14).

This parallelism between cavity couplings and allosteric ones suggests a possible mechanism for the origin of allostery. At present it is still unclear how allostery appeared along protein evolution[100,101]. Liang et al.[100] divide this process into two components: appearance of the coupling between the main functional site and the allosteric site, followed by the creation of effector affinity in the allosteric site through a series of mutagenic events. After underlining how unlikely is it for effector affinity to evolve, these authors propose that ligand binding and allostery may have appeared simultaneously. Our results suggest a simpler alternative, in which the need for allosteric coupling creation could be eliminated, since one of the several spontaneous couplings between the main cavity and a secondary cavity could act as a seed. Allostery appearance would only require the creation of effector-binding properties in this cavity.

## Conclusion

We have explored and characterized the occurrence of cavity couplings in the dynamics of proteins around the native structure. To this end we have used 2x20 ns MD simulations for 75 proteins, and ≥100 ns MD simulations from the MoDEL project for 41 proteins. In the first case, after applying a stringent filtering procedure we obtained a set of 297 CSCs distributed over 60% of the proteins in our dataset, and identified some of the structural factors contributing to such couplings. This result was extended using the ≥100 ns simulations. We found that correlations between cavities may vary with time (Fig. 14) and that the overall fluctuation pattern of these correlations is comparable between proteins (Fig. 15). However, some proteins show less persistent couplings than others, and cavity neighbourhood naturally also plays a role in biasing correlations towards higher values (Fig. 16). The results are robust to variations in simulation length (Fig. 15B and 15D), since they were similar for 100 ns simulations and five longer simulations (S2 Table). Finally, we discuss some of the biological implications of our results.

## Supporting Information

S1 Fig The effect of using different moving window sizes on the results in Fig. 15. Five different sizes (10, 20, 30, 40, 50) were tried to reproduce the results in Fig. 15 for lysozyme (PDB code: 153l). The results for n = 20 are those plotted in Fig. 15; we see no difference for the results obtained with the other window sizes.
(TIF)

S2 Fig Relationship between correlation size and protein divergence. This figure is analogous to Fig. 12, except that in this case we have used Kimura's distance as a measure of sequence divergence, instead of the percentage of sequence identity or crmsd. The procedure followed here was the same as in Fig. 12. For each of the 14 pairs of couplings conserved between species, we computed the average between the human and the other species correlation (an average of two values). Then, we plotted the resulting 14 values as a function of Kimura's distance. We saw no relationship suggesting that Kimura's distance is related to correlation conservation.
(TIF)

S1 Table List of the proteins in the D75 set. In the first and second columns we provide the PDB codes of the human protein and that of its ortholog, respectively. In the third column we provide the protein name. In the fourth and fifth columns, we provide the CATH class and architecture, respectively.
(DOCX)

S2 Table List of the proteins in the D41 set. In the first column we provide the PDB code of the protein. In the second column we provide the protein name. In the third and fourth columns, we provide the CATH class and architecture, respectively. In the fifth column, we provide the simulation length (ns).
(DOCX)

S3 Table List of human lysozyme mutants used in this work. In the first column we provide the PDB code of the mutant, and in the second column we describe the mutation.
(DOC)
