# Supplementary figures and images for: Molecular Dynamics Study of Naturally Existing Cavity Couplings in Proteins
Source: PLoS One. 2015 Mar 27;10(3):e0119978. doi: 10.1371/journal.pone.0119978 (PMC4376744; doi:10.1371/journal.pone.0119978)

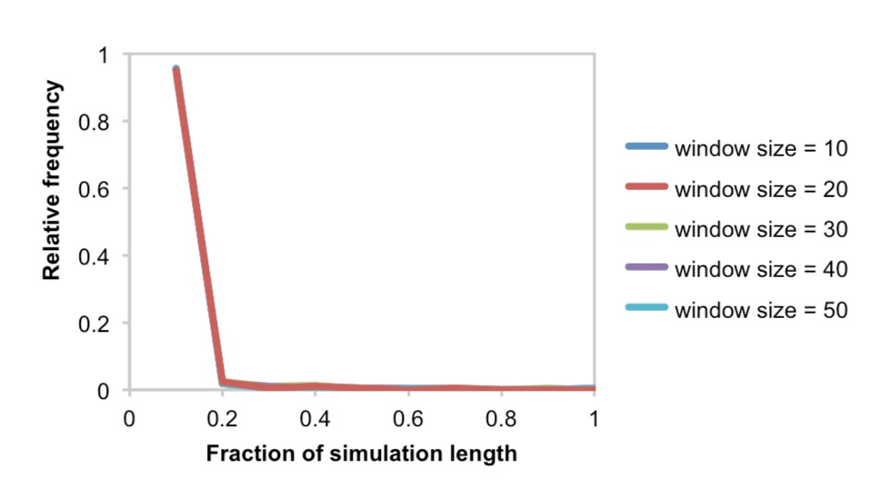

Supplement: S1 Fig — Five different sizes (10, 20, 30, 40, 50) were tried to reproduce the results in Fig. 15 for lysozyme (PDB code: 153l). The results for n = 20 are those plotted in Fig. 15; we see no difference for the results obtained with the other window sizes. (TIF) [file pone.0119978.s001.tif]

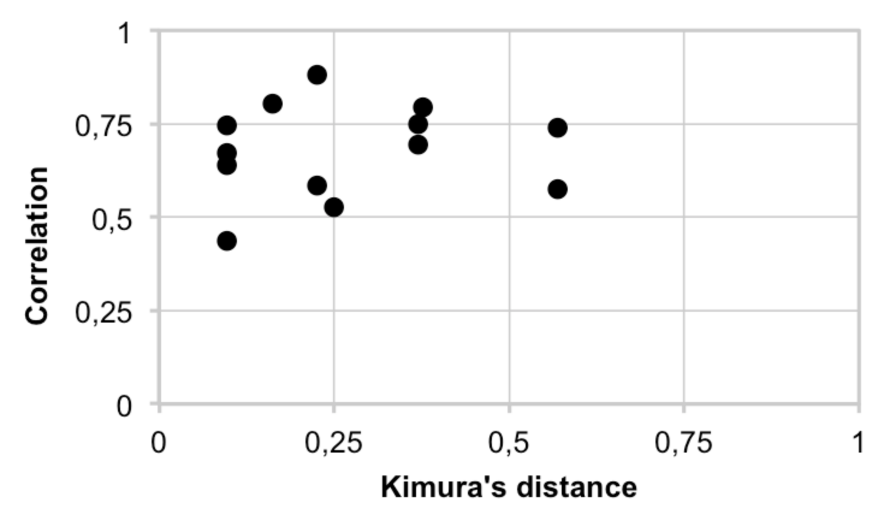

Supplement: S2 Fig — This figure is analogous to Fig. 12, except that in this case we have used Kimura's distance as a measure of sequence divergence, instead of the percentage of sequence identity or crmsd. The procedure followed here was the same as in Fig. 12. For each of the 14 pairs of couplings conserved between species, we computed the average between the human and the other species correlation (an average of two values). Then, we plotted the resulting 14 values as a function of Kimura's distance. We saw no relationship suggesting that Kimura's distance is related to correlation conservation. (TIF) [file pone.0119978.s002.tif]
