# Supplementary material for: Molecular Dynamics Study of Naturally Existing Cavity Couplings in Proteins
Source: PLoS One. 2015 Mar 27;10(3):e0119978. doi: 10.1371/journal.pone.0119978 (PMC4376744; doi:10.1371/journal.pone.0119978)
Supplement: S1 Table — In the first and second columns we provide the PDB codes of the human protein and that of its ortholog, respectively. In the third column we provide the protein name. In the fourth and fifth columns, we provide the CATH class and architecture, respectively. (DOCX) [file pone.0119978.s003.docx]

**S1 Table. Set of 75 proteins simulated in this work (2x20 ns MD simulations)**

| **HUMAN** | **ORTHOLOG** | **PROTEIN NAME** | **CATH**  **(Class)** | **CATH**  **(Arquitecture)** |
| --- | --- | --- | --- | --- |
| 1a4v | 1f6s | alpha-lactalbumin | Mainly Alpha | Orthogonal bundle |
| 1ang | 1agi | angiogenin | Alpha Beta | Roll |
| 1ang | 2bwk | angiogenin | Alpha Beta | Roll |
| 1anx | 1yii | annexin V | Mainly Alpha | Orthogonal bundle |
| 1anx | 2ran | annexin V | Mainly Alpha | Orthogonal bundle |
| 1auc | 1xw9 | thioredoxin | Alpha Beta | 3-Layer (aba) Sandwich |
| 1bp5 | 1tfa | transferrin | Alpha Beta | 3-Layer (aba) Sandwich |
| 1brq | 1hbq | retinol-binding protein | Mainly Beta | Beta barrel |
| 1hup | 1rdo | mannose-binding protein | Alpha Beta | Roll |
| 1ime | 2bji | inositol monophosphatase | Alpha Beta | 2-Layer Sandwich |
| 1iu1 | 1gyu | gamma-adaptin | Mainly Beta | Sandwich |
| 1j7d | 1jbb | ubc13 | Alpha Beta | Roll |
| 1jxv | 1ndl | nucleoside diphosphate kinase | Alpha Beta | 2-Layer Sandwich |
| 1kfu | 1aj5 | calpain | Mainly Alpha | Orthogonal bundle |
| 1kfu | 1nx2 | calpain | Mainly Alpha | Orthogonal bundle |
| 1kpb | 6rhn | kinase C interacting protein | Alpha Beta | 2-Layer Sandwich |
| 1kps_1 | 1u9a | ubiquitin conjugating enzyme ubc9 | Alpha Beta | Roll |
| 1m73 | 1pbn | purine nucleoside phosphorylase | Alpha Beta | 3-Layer (aba) Sandwich |
| 1mb8 | 1sh6 | plectin | Mainly Alpha | Orthogonal bundle |
| 1psn | 5pep | pepsin | Mainly Beta | Beta barrel |
| 1shf | 1z9z | tyrosine kinase SH3 domain | Mainly Beta | Roll |
| 1tbt | 1v9e | carbonic anhidrase II | Alpha Beta | Roll |
| 1u6d | 1x2j | keap1 | Mainly Beta | 6 Propellor |
| 1ubq | 1aar | ubiquitin | Alpha Beta | Roll |
| 1uoh | 1ixv | gankyrin | Mainly Alpha | Alpha Horseshoe |
| 1w08 | 1dpx | lysozyme | Mainly Alpha | Orthogonal bundle |
| 1wm2 | 1euv | ubiquitin-like protein SMT3B | Alpha Beta | Roll |
| 1wyi | 8tim | triose phosphate isomerase | Alpha Beta | Alpha-Beta Barrel |
| 1yes | 1ah6 | heat shock protein HSP90 | Alpha Beta | 2-Layer Sandwich |
| 1yla | 2bf8 | ubiquitin conjugating enzyme E2 | Alpha Beta / Mainly Alpha | Roll / Orthogonal bundle |
| 1yla | 1fzy | ubiquitin conjugating enzyme E2 | Alpha Beta / Mainly Alpha | Roll / Orthogonal bundle |
| 1yyh | 1ot8 | notch | Mainly Alpha | Alpha Horseshoe |
| 1yyh | 1ymp | notch | Mainly Alpha | Alpha Horseshoe |
| 2a8b | 1jln | tyrosine phosphatase Receptor | Alpha Beta | Alpha-Beta complex |
| 2ald | 1zah | aldolase | Alpha Beta | Alpha-Beta Barrel |
| 2ald | 1fba | aldolase | Alpha Beta | Alpha-Beta Barrel |
| 2bz8 | 1zx6 | sh3 domain kinase binding protien | Mainly Beta | Roll |
| 2cpl | 1dyw | cyclophilin A | Mainly Beta | Beta barrel |
| 2cpl | 1ist | cyclophilin A | Mainly Beta | Beta barrel |
| 2grn | 1kps_2 | ranGAP1 | Mainly Alpha | Alpha Horseshoe |
| 4i1b | 8i1b | interleukin-1 beta | Mainly Beta | Trefoil |
