# Supplementary material for: Molecular Dynamics Study of Naturally Existing Cavity Couplings in Proteins
Source: PLoS One. 2015 Mar 27;10(3):e0119978. doi: 10.1371/journal.pone.0119978 (PMC4376744; doi:10.1371/journal.pone.0119978)
Supplement: S2 Table — In the first column we provide the PDB code of the protein. In the second column we provide the protein name. In the third and fourth columns, we provide the CATH class and architecture, respectively. In the fifth column, we provide the simulation length (ns). (DOCX) [file pone.0119978.s004.docx]

**S2 Table. Set of 41 proteins retrieved from the MoDEL project.**

| **PDB CODE** | **PROTEIN NAME** | **CATH (Class)** | **CATH**  **(Arquitecture)** | **MD SIMULATION LENGTH (ns)** |
| --- | --- | --- | --- | --- |
| 1agi | Angiogenin | Alpha Beta | Roll | 100 |
| 1ark | Nebulin SH3 domain | Mainly Beta | Roll | 100 |
| 1bfg | Bovine lipocalin allergen Bos D2 | Mainly Beta | Trefoil | 100 |
| 1bj7 | Basic fibroblast growth factor | Mainly Beta | Beta Barrel | 100 |
| 1bpi | Pancreatic trypsin inhibitor | Few secondary structures | Irregular | 100 |
| 1bsn | F1-Atpsynthase (epsilon subunit) | Mainly Beta / Mainly Alpha | Sandwich / Up-Down Bundle | 100 |
| 1cbs | Cellular retinoic-acid binding protein | Mainly Beta | Beta Barrel | 100 |
| 1cei | Colicin E7 immunity protein | Mainly Alpha | Orthogonal Bundle | 100 |
| 1chn | Chemotaxis protein Chey | Alpha Beta | 3-Layer (aba) Sandwich | 100 |
| 1csp | Cold shock protein (CSPB) | Mainly Beta | Beta Barrel | 100 |
| 1fas | Fasciculin 1 | Mainly Beta | Ribbon | 100 |
| 1fvq | Copper-Transporting domain CCC2A | Alpha Beta | 2-Layer Sandwich | 100 |
| 1i6f | Neurotoxin V-5 | Alpha Beta | 2-Layer Sandwich | 100 |
| 1j5d | Plastocyanin | Mainly Beta | Sandwich | 100 |
| 1jli | Interleukin 3 | Mainly Alpha | Up-down Bundle | 100 |
| 1jw2 | Hemolysin expression modulating protein Hha | Mainly Alpha | Up-down Bundle | 100 |
| 1k40 | Focal adhesion kinase | Mainly Alpha | Up-down Bundle | 100 |
| 1kxa | Sindbis virus capsid protein | Mainly Beta | Beta Barrel | 100 |
| 1lit | Lithostathine | Alpha Beta | Roll | 100 |
| 1lki | Leukemia inhibitory factor | Mainly Alpha | Up-down Bundle | 100 |
| 1ls9 | Cytochrome C6 | Mainly Alpha | Orthogonal Bundle | 100 |
| 1lys | Hen egg-white lysozyme | Mainly Alpha | Orthogonal Bundle | 100 |
| 1nso | Protease | Mainly Beta | Beta Barrel | 100 |
| 1ooi | Alcohol binding protein LUSH | Mainly Alpha | Orthogonal Bundle | 100 |
| 1pdo | Mannose permease | Alpha Beta | 3-Layer (aba) Sandwich | 100 |
| 1pgb | Protein G | Alpha Beta | Roll | 100 |
| 1pht | Phosphatidylinositol 3-kinase | Mainly Beta | Roll | 100 |
| 1sdf | Stromal cell-derived factor-1 | Mainly Beta | Beta Barrel | 100 |
| 1sp2 | SP1F2 | - | - | 100 |
| 1sro | Pnpase | Mainly Beta | Beta Barrel | 100 |
| 1sur | Phospho-adenylyl-sulfate reductase | Alpha Beta | 3-Layer (aba) Sandwich | 100 |
| 1txa | Toxin B | Mainly Beta | Ribbon | 100 |
| 2hvm | Hevamine | Alpha Beta | Alpha-Beta Barrel | 100 |
| 3ci2 | Chymotrypsin inhibitor 2 | Alpha Beta | 2-Layer Sandwich | 100 |
| 4icb | Calbindin D9K | Mainly Alpha | Orthogonal Bundle | 100 |
| 153l | Goose Lysozyme | Mainly Alpha | Orthogonal Bundle | 100 |
| 1cqy | Beta-amylase | Mainly Beta | Sandwich | 769 |
| 1kte | Thioltransferase | Alpha Beta | 3-Layer (aba) Sandwich | 998 |
| 1opc | OMPR | Mainly Alpha | Orthogonal Bundle | 791 |
| 1ubq | Ubiquitin | Alpha Beta | Roll | 1000 |
| 2gb1 | Protein G | Alpha Beta | Roll | 1000 |
