# Supplementary material for: Molecular Dynamics Study of Naturally Existing Cavity Couplings in Proteins
Source: PLoS One. 2015 Mar 27;10(3):e0119978. doi: 10.1371/journal.pone.0119978 (PMC4376744; doi:10.1371/journal.pone.0119978)
Supplement: S3 Table — In the first column we provide the PDB code of the mutant, and in the second column we describe the mutation. (DOC) [file pone.0119978.s005.doc]

**S3 Table. List of human lysozyme mutants used in this work.**
